# Analysis of Clinically Symptomatic Patients to Differentiate Inflammatory Breast Cancer from Mastitis in Asian Women

**DOI:** 10.3390/life15010005

**Published:** 2024-12-24

**Authors:** Han Song Mun, Ha Yeun Oh

**Affiliations:** 1 Department of Radiology, Seoul St. Mary’s Hospital, College of Medicine, The Catholic University of Korea, Seoul 06591, Republic of Korea; im_hsm@catholic.ac.kr; 2 Department of Radiology, College of Medicine/School of Medicine, Kangwon National University, Chuncheon-si 24341, Republic of Korea; 3 Department of Radiological Sciences, University of California, Irvine, CA 92697, USA

**Keywords:** inflammatory breast cancer, mastitis, ultrasound, Asian

## Abstract

Purpose: To differentiate inflammatory breast cancer (IBC) from mastitis in Asian women presenting with symptoms of inflammation. Methods: Between January 2012 and June 2024, 101 Asian women with symptoms of inflammation underwent breast ultrasound (US). Clinical and demographic data were extracted from patients’ medical records. US analysis assessed lesion bilaterality, location, type, size, internal changes, and lymph node status. Patients with suspicious findings had US-guided biopsies, and pathology reports were reviewed for tumor histology and immunohistochemical markers. Logistic regression was used to determine odds ratios. Results: Of the 101 participants, 14 (13.9%) were diagnosed with IBC and 87 (86.1%) were diagnosed with mastitis. Patients with IBC were significantly older (46.4 vs. 38.4 years, *p* = 0.020) and showed a higher prevalence of postmenopausal status (57.1% vs. 12.6%, *p* < 0.0001). These patients experienced a longer symptom onset duration (37.7 vs. 12.7 days, *p* = 0.002) and more frequent localized symptoms like swelling (50.0% vs. 13.8%, *p* = 0.004). US findings showed that 21.4% of IBC lesions involved the entire breast, compared to only 1.1% in patients with mastitis (*p* = 0.001). Biopsy results revealed that invasive ductal carcinoma was the most common malignancy (78.6%). Logistic regression identified symptom onset duration (adjusted odds ratio (OR) 1.07, *p* = 0.014) and swelling (adjusted OR 15.24, *p* = 0.016) as significant predictors of IBC. Conclusion: In Asian women, age, menopausal status, symptom onset duration, and swelling are effective in differentiating IBC from mastitis. Logistic regression confirmed that symptom onset duration and swelling are significant predictors of IBC, with US findings indicating larger lesion sizes and more frequent whole-breast involvement.

## 1. Introduction

Inflammatory breast, sometimes referred to as red breast syndrome, is a classic but rare complaint among women in both primary care settings and dedicated breast centers [1,2]. It typically manifests as redness and warmth of the breast and is frequently accompanied by breast pain [3,4]. The spectrum of possible diagnoses for women presenting with inflammatory breast symptoms is broad, ranging from benign, self-limited conditions to progressive, malignant diseases [1]. The malignant form of this condition, known as inflammatory breast cancer (IBC), has a high risk of metastasis and recurrence [5]. Lee and Tannebaum first described inflammatory carcinoma of the breast in 1924 [6]. In the United States, IBC is estimated to account for approximately 2–4% of new breast cancer diagnoses annually, yet it is responsible for 7–10% of breast cancer-related deaths [7].

Based on an estimated incidence of 125 breast cancer cases per 100,000 women, the incidence of IBC is approximately 2–3 cases per 100,000 women [8]. Consequently, many generalists may never encounter IBC during their careers. Studies indicate that among women presenting with an inflamed breast, the incidence of IBC ranges from 5–50%, underscoring its relevance in cases of breast inflammation, particularly in non-pregnant or non-postpartum patients [1,9,10].

All IBC studies, with the exception of a single study from France [9], have been conducted in the United States [1,11,12,13,14] and have generally excluded Asian women from their analyses. Additionally, original research has included cohorts ranging from 22–76 participants [1,9]. Jagsi et al. [14] proposed a quantitative scoring system to distinguish IBC from non-inflammatory locally advanced breast cancer based on clinical, pathological, and imaging features.

Breast cancer constitutes 24.5% of all cancers in female patients, with nearly half of breast cancer patients (45.4%) diagnosed in Asia [15]. Breast cancer tends to affect Asian women at an earlier age compared to Western women, with peak incidence occurring between 40 and 50 years in Asian countries and between 60 and 70 years in Western countries [16,17].

Therefore, this study aimed to investigate and differentiate IBC from mastitis in Asian women presenting with inflammatory symptoms. Our study included women of all ages, both postpartum and non-postpartum, in a large consecutive cohort.

## 2. Materials and Methods

### 2.1. Study Population

**Figure 1:**
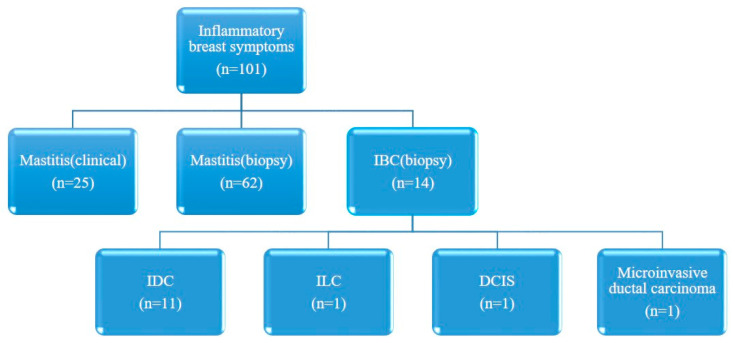
Schematic diagram for the inclusion of participants.

This study was approved by our Institutional Review Board (IRB No. 2024-07-009), and the requirement for informed consent was waived due to its retrospective nature. The study adhered to the principles outlined in the Declaration of Helsinki. From January 2012 to June 2024, a total of 118 women presenting with inflammatory breast symptoms underwent breast ultrasound (US) at our facility. Nine patients were excluded owing to incomplete demographic and clinical information, and eight patients were excluded owing to poor US image quality or the removal of images from data storage. Consequently, 101 participants were included in the study; among them, 25 were diagnosed with clinical mastitis, 62 underwent biopsy for mastitis, and 14 had biopsies confirming IBC (Figure 1).

All study participants underwent initial evaluation for inflammatory breast symptoms at the time of diagnosis and were not receiving any treatment.

IBC denotes inflammatory breast cancer; IDC denotes invasive ductal carcinoma; ILC denotes invasive lobular carcinoma; and DCIS denotes ductal carcinoma in situ.

### 2.2. Clinical and Demographic Data

Clinical and demographic data were collected, including age, body mass index (BMI, weight/height^2^) [18], pregnancy or lactation status, menopause status, history of mastitis, personal history of breast or other cancers, and underlying conditions such as diabetes, metabolic syndrome, or immunocompromised state. Information on current regular alcohol consumption and smoking habits was also gathered from the patients’ electronic medical records. For breast-related aspects, data on inflammatory symptoms included duration of symptom onset, types of localized symptoms (erythema, swelling, skin changes, warmth, palpable mass/lesion, and pain/discomfort), and systemic symptoms (fever, malaise, myalgia, flu-like symptoms, or none).

### 2.3. Ultrasound Examination and Analysis

Two breast radiologists, with 13 and 15 years of experience in breast imaging, conducted preoperative US examinations. Images were obtained using various high-resolution US units, including the ATL HDI 5000 (Philips-Advanced Technology Laboratories, Bothell, WA, USA) equipped with a 12–5 MHz linear array transducer, the iU22 (Philips Healthcare, Bothell, WA, USA) with a 12–5 MHz linear array transducer, and the Epic-7 and Epic Elite (Philips Healthcare, Bothell, WA, USA) with an eL18-4 MHz linear array transducer.

All patients underwent bilateral breast and axillary examinations. US images were analyzed in consensus by the radiologists using a INFINITT PACS system (Version: 3.0.11, INFINITT Healthcare, Seoul, Republic of Korea). The findings documented included bilaterality of the lesions, whether they were located in the right breast, left breast, or both, lesion type, and whether they were masses or non-mass formations. The location of the lesions was noted as central or peripheral, and their focality was classified as either single or multiple lesions. Echogenicity was assessed as hypoechoic, isoechoic, or hyperechoic, while the echo pattern was determined to be either homogeneous or heterogeneous. The size of the lesions was evaluated in relation to the whole breast, with categories including less than half, the same or larger than half, or involving the entire breast.

Additionally, internal changes within the lesions, such as cystic or necrotic alterations, were noted, as well as the presence or absence of lesion vascularity, categorized as minimal/mildly increased, moderate-to-severely increased, or none. Other findings included the presence of skin thickening, parenchymal edema, nipple inversion, calcification, and ductal changes. The status of the axillary lymph nodes was also assessed, with nodes being classified as enlarged, equivocal, or within the normal range.

### 2.4. Histopathology Review

After undergoing US examination, selected patients recommended by clinicians or radiologists underwent US-guided biopsy to confirm the diagnosis of mastitis and exclude other diseases, especially malignancy. Pathology reports were reviewed to determine tumor histology. Additional information regarding tumor grade and immunohistochemical (IHC) subtypes was collected from the patients’ electronic medical records after breast malignancy was confirmed. The following biomarkers were evaluated via IHC: estrogen receptor (ER), progesterone receptor (PR), human epidermal growth factor receptor 2 (HER2), and Ki-67. IHC staining was performed using an automated Ventana BenchMark XT Slide Stainer (Ventana, Tucson, AZ, USA). Tumor subtypes were classified as hormone receptor (HR) positive/HER2 negative, HR positive/HER2 positive, HR negative/HER2 positive, or HR negative/HER2 negative (triple-negative). Positive Ki-67 expression was defined as Ki-67 positivity in ≥20% of cancer cell nuclei.

### 2.5. Statistical Analysis

Clinical and demographic data of study participants were analyzed by calculating the mean, standard deviation, and numbers (%). To determine the distribution differences between IBC and mastitis for categorical variables, Fisher’s Exact Test or the Pearson Chi-Square Test was employed. For assessing mean differences in continuous variables, we performed a *t*-test. Additionally, logistic regression was used to calculate the crude odds ratio (OR), and an analysis was conducted to determine the adjusted OR, evaluating the impact of differences between IBC and mastitis. All statistical analyses were performed using SAS version 9.4 (SAS Institute Inc., Cary, NC, USA), and two-tailed *p*-values < 0.05 were considered to be statistically significant.

## 3. Results

### 3.1. Clinical and Demographic Characteristics

The study included 101 participants, with 14 (13.9%) diagnosed with IBC and 87 (86.1%) diagnosed with mastitis (Table 1). Patients with IBC had a significantly higher mean age of 46.4 years compared to 38.4 years in the mastitis group (*p* = 0.020). Additionally, a greater proportion of patients with IBC were aged 40 years or older (78.6%) compared to those with mastitis (26.4%) (*p* < 0.001).

Body mass index (BMI) also differed significantly between the groups. Patients with IBC had a higher mean BMI of 28.8 compared to 24.4 in the mastitis group (*p* = 0.009). All patients were either overweight or obese, with 87.5% categorized as obese; only 53.5% of patients fell into these categories (*p* = 0.012).

Menopausal status was significantly associated with IBC. Approximately 57.1% of patients with IBC were postmenopausal, compared to only 12.6% of patients with mastitis (*p* < 0.0001). The onset of symptoms was longer in patients with IBC, with a mean duration of 37.7 days compared to 12.7 days for patients with mastitis (*p* = 0.002). Localized symptoms such as swelling were more common in patients with IBC (50.0%) than in those with mastitis (13.8%) (*p* = 0.004). Other localized or systemic symptoms did not differ significantly between the groups. However, a history of previous mastitis was significantly less common in patients with IBC (7.1%) compared to those with mastitis (37.9%) (*p* = 0.019).

### 3.2. Ultrasound Features

**Figure 2:**
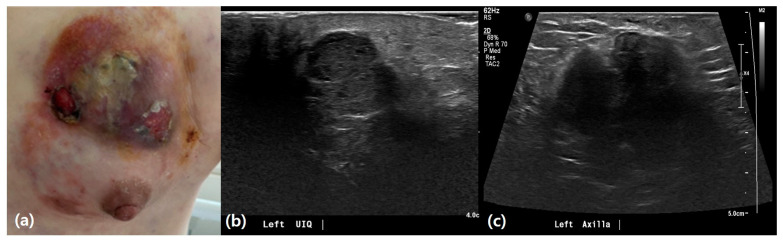
Inflammatory breast cancer. A 40-year-old female patient presented with a palpable mass and skin changes in her left breast. (**a**) Clinical appearance showing erythema, skin thickening, and ulcerative changes at the time of the visit. (**b**) Ultrasound imaging demonstrates an irregular hypoechoic mass with diffuse skin thickening and edematous changes, primarily in the left upper inner quadrant. (**c**) Ultrasound imaging also shows a conglomerated lymph node, approximately 4.7 cm in size, in the left axillary level I, suggestive of metastatic involvement.

**Figure 3:**
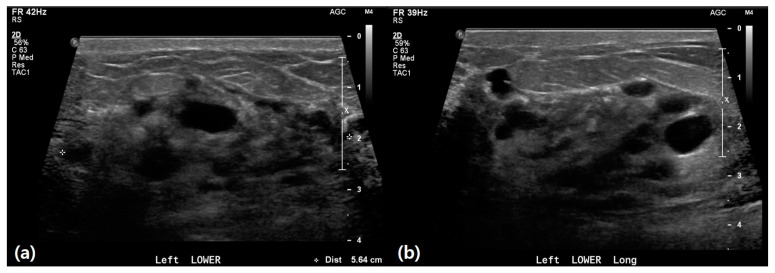
A 43-year-old female presented with erythema, a palpable mass, and pain in the breast. (**a**,**b**) Ultrasound imaging revealed heterogeneous echo texture in both breasts. The lower portion of the left breast exhibited diffuse skin thickening and edematous changes, with an indistinct heterogeneous hypoechoic lesion containing cystic components. These findings suggest a high likelihood of an infectious condition, such as mastitis. The patient was treated with medication, and clinical follow-up indicated improvement in symptoms.

US findings revealed significant differences between patients with IBC and those with mastitis (Table 2). IBC lesions were notably larger, with 21.4% involving the whole breast versus 1.1% in mastitis (*p* = 0.001) (Figure 2). Cystic/necrotic changes were less common in IBC (7.1%) compared to mastitis (33.3%) (*p* = 0.039) (Figure 3). Although the difference was not statistically significant, patients with IBC had a higher frequency of non-mass lesions (78.6%) and centrally located lesions (61.5%) compared with those with mastitis (67.8% and 38.8%, respectively). This group also exhibited more minimal to mild vascularity and skin thickening (85.7% and 92.9%, respectively) than patients with mastitis (59.8% and 80.5%). Parenchymal edema was present in all patients with IBC, compared to 86.2% of patients with mastitis. Additionally, patients with IBC had slightly higher rates of nipple inversion (28.6%) and calcifications (7.1%) compared to patients with mastitis (14.9% and 2.3%). Ductal changes and lymphadenopathy were more common in patients with IBC (35.7% and 57.1%) than in those with mastitis (32.2% for both), although these differences were not statistically significant.

### 3.3. Pathological and Biopsy Results

**Figure 4:**
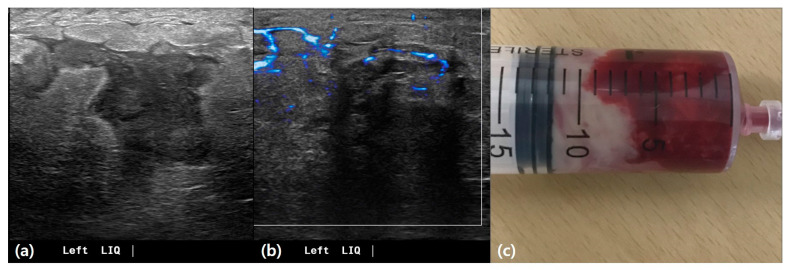
Idiopathic granulomatous mastitis with abscess. A 35-year-old female patient presented with breast discomfort persisting for 14 days. (**a**) Ultrasound imaging demonstrates a hypoechoic lesion with diffuse skin thickening and edematous changes in the lower inner quadrant of the left breast. (**b**) Doppler ultrasound imaging reveals a moderate increase in blood flow, shown in blue. (**c**) Ultrasound-guided needle aspiration was performed, and 12 cc of bloody pus was aspirated.

Among benign mastitis cases (*n* = 62), the most common finding was chronic granulomatous inflammation (50.0%) (Figure 4), followed by inflammation with abscess formation (37.1%). For malignant biopsies (*n* = 14), invasive ductal carcinoma was predominant (78.6%), with one case each of microinvasive ductal carcinoma, invasive lobular carcinoma, and ductal carcinoma in situ (each 7.1%) (Table 3).

Among the 13 invasive cancer cases found in US-guided biopsies, 38.5% were moderately differentiated and 61.5% were poorly differentiated. Estrogen receptor positivity was found in 53.8%, while progesterone receptor positivity was found in 46.2%. HER2 positivity was observed in 61.5% of tumors. The Ki-67 proliferation index exceeded 20% in 61.5% of cases, indicating high tumor aggressiveness. The tumor subtypes were HR+/HER2+ (38.5%), HR-/HER2+ (23.1%), HR-/HER2- (23.1%), and HR+/HER2- (15.4%) (Table S1).

### 3.4. Logistic Regression Analysis of Factors Associated with IBC

Table 4 presents the logistic regression analysis of factors associated with IBC. Statistically significant variables from clinical and demographic data, as well as US findings, were used for this analysis, as shown in Table 1 and Table 2. The crude analysis identified age, BMI, menopausal status, symptom onset duration, breast swelling, and lesion size as significant predictors. However, BMI was excluded from the adjusted model owing to potential data issues suggested by an extremely high crude OR. After adjusting for other variables, symptom onset duration and breast swelling emerged as clinically significant predictors of inflammatory breast cancer. The adjusted OR for symptom onset duration was 1.07 (95% CI: 1.02–1.14) with a *p*-value of 0.014, and breast swelling had an adjusted OR of 15.24 (95% CI: 1.68–138.69) with a *p*-value of 0.016.

## 4. Discussion

We analyzed 101 Asian women presenting with inflammatory symptoms; of them, 14 (13.9%) were diagnosed with IBC and 87 (86.1%) were diagnosed with mastitis. In 2009, Kamal et al. [10] found that 5.6% of patients had IBC in a cohort of 197 patients with mastitis. Similarly, Froman et al. [1] reported a rate of 4.5% of IBC in a cohort of 23 patients presenting with red breast syndrome after screening more than 3700 women at their breast unit over a two-year period. In another report [9], non-pregnant or postpartum women presenting with inflammatory breast symptoms had a breast cancer incidence of 50%. Our study included participants aged 22–82, both postpartum and non-postpartum, and found that the incidence of IBC in non-postpartum cases was 16.7% (13/78), which is within the range of previous studies.

The diagnostic criteria for IBC include typical symptoms such as erythema occupying more than one-third of the breast, edema, peau d’orange, and warmth. These symptoms may or may not be associated with an underlying palpable mass [12]. The time from onset to full presentation is usually within three months and never more than six months, which distinguishes IBC from locally advanced non-IBC. In our study, the most common presenting symptoms among patients diagnosed with IBC included swelling and palpable mass (50% each), pain/discomfort (42.9%), and erythema (35.7%). A retrospective review identified erythema (62%), edema or fullness (48%), and skin dimpling or discoloration (46%) as the most common presenting clinical signs. However, Lê et al. [19] found that, in most patients with IBC, no discrete mass is palpable on clinical examination. This may be because breast enlargement due to swelling may cause confusion.

Our findings indicate that older age, higher BMI, postmenopausal status, longer symptom duration, and absence of previous mastitis history are associated with an increased likelihood of IBC compared to mastitis. Dabi et al. [9] reported that patients with malignant lesions were significantly older and had a significantly larger palpable mass compared to those with benign disease. Another study identified high BMI as one of the strongest risk factors for IBC [13]. These findings are consistent with our results. Although there is no report on symptom onset duration, patients with IBC statistically had a longer duration of symptoms than those with mastitis in our study.

Despite multimodal treatment approaches, survival outcomes for IBC remain poor compared to matched non-inflammatory controls [20]. Historical data estimate that the 5- and 10-year overall survival rates for stage 3 IBC are between 40–45% and 30–35%, respectively [21,22,23]. Due to the aggressive nature of IBC, early diagnosis is crucial. Routine screening mammography is not effective in the early detection of IBC and is the least sensitive among breast imaging modalities [22].

For women at average risk of breast cancer, one study demonstrated that adding US to mammography detected more cases of breast cancer during screening [24]. In women with dense breasts, cohort studies more reflective of real-life clinical settings confirmed these results, while studies on women with non-dense breasts showed no statistically significant differences between the two modalities. Regarding neoadjuvant tumor size prediction, another study suggested that both mammography and breast US outperformed clinical palpation [25]. While mammography was slightly more accurate than US in estimating the exact tumor size, the difference was not statistically significant. Notably, 29 out of 193 (15%) tumors were assessable solely by breast US.

In patients with symptoms but no precise initial diagnosis, bilateral breast and nodal USs may be helpful. Our US findings suggest that larger lesion size and absence of cystic/necrotic changes are important characteristics that may help differentiate IBC from mastitis. Other studies have revealed that the presence of precise limits of the mass is only associated with IBC [9,10,26].

Although published data on MRI findings in patients with IBC are limited, MRI is the most accurate test for detecting a primary breast lesion in these patients [27]. For contrast-enhanced MRI, non-mass-like enhancement, rapid initial enhancement with a washout pattern, and diffuse cutaneous/subcutaneous and prepectoral edema on T2-weighted images are key indicators of IBC [28]. However, MRI is not easily accessible in our daily practice for patients presenting with inflammatory symptoms before undergoing US examinations.

Pathologic analysis should confirm invasive breast carcinoma. IBC is often associated with the infiltration of dermal lymphatics by tumor emboli, accounting for the characteristic edema and skin changes. However, this is not required for diagnosis nor always seen in biopsy specimens. In our study, invasive ductal carcinoma was predominant (78.6%). Previous studies have revealed that lobular histologic characteristics are less common in IBC than in non-IBC cases [29]. However, our study did not include lobular histology, possibly due to its small sample size. Among biopsy-proven benign pathologies, 47.1% showed abscess formation or granulomatous inflammation, which may have affected US findings suggestive of cystic/necrotic changes.

To the best of our knowledge, no previous studies have used regression analysis to identify the impact of variables in distinguishing IBC from mastitis. Logistic regression analysis identified symptom onset duration and breast swelling as clinically significant predictors of IBC, even after adjusting for other variables. The adjusted OR for symptom onset duration was 1.07, and for breast swelling, it was 15.24, both indicating strong associations with IBC.

Our study has some limitations that should be acknowledged. First, like other retrospective analyses, there is a risk of selection bias and potential inaccuracies in chart data extraction. Similarly, the granularity of the extracted electronic medical records was limited. Second, we did not analyze mammography findings. Mammography is generally less specific in distinguishing between mastitis and IBC [30], especially since our study included post-partum and young patients. This limitation is consistent with the study’s primary focus on utilizing US as the main diagnostic tool. Third, due to the rarity of IBC, the number of patients we studied was small despite our relatively large study population. This may have limited our ability to detect subtle differences between IBC and mastitis. However, one of the strengths of our study is the inclusion of consecutive Asian women over a 12-year period. Finally, due to prompt referral to an academic tertiary care center with an established multidisciplinary clinic for the treatment of IBC upon diagnosis, our study did not include details on the treatment course or surgical outcomes of patients.

In conclusion, age, BMI, menopausal status, symptom onset duration, and breast swelling significantly differentiated IBC from mastitis in Asian women. Logistic regression identified symptom onset duration and breast swelling as significant IBC predictors. US findings showed larger lesion size and more frequent whole-breast involvement in patients.

## Figures and Tables

**Table 1:** Clinical and demographic data of the study participants.

Variables	Total	IBC	Mastitis	*p*-Value
Number of Patients	101	*n* = 14 (13.9%)	*n* = 87 (86.1%)	
Age				
(mean ± SD)	39.5 ± 12.1	46.4 ± 11.4	38.4 ± 11.9	0.020
(median, IQR)	37.0 (32.0–44.0)	44.5 (39.8–58.3)	35.0 (32.0–4.0)	
≥40 years	34 (33.7%)	11 (78.6%)	23 (26.4%)	<0.001
<40 years	67 (66.3%)	3 (21.4%)	64 (73.6%)	
BMI				
(mean ± SD)	25.1 ± 4.5	28.8 ± 3.9	24.4 ± 4.3	0.009
Underweight: BMI < 18.5	0 (0.0%)	0 (0.0%)	0 (0.0%)	0.043
Normal weight: BMI 18.5–22.9	20 (39.2%)	0 (0.0%)	20 (46.5%)	
Overweight: BMI 23–24.9	5(9.8%)	1(12.5%)	4 (9.3%)	
Obesity: BMI ≥ 25	26 (51.0%)	7(87.5%)	19 (44.2%)	
Normal weight	20 (39.2%)	0(0.0%)	20 (46.5%)	0.012
Overweight + Obesity	31 (60.8%)	8 (100.0%)	23 (53.5%)	
Menopausal Status				
Premenopause	82 (81.2%)	6 (42.9%)	76 (87.4%)	<0.0001
Postmenopause	19 (18.8%)	8 (57.1%)	11 (12.6%)	
During Pregnancy/Lactation				
Yes	23 (22.8%)	2 (14.3%)	21 (24.1%)	0.333
No	78 (77.2%)	12 (85.7%)	66 (75.9%)	
Symptom Onset (days ago)				
(mean ± SD)	16.7 ± 17.0	37.7 ± 21.3	12.7 ± 12.8	0.002
(median, IQR)	14.0 (7.0–30.0)	60.0 (18.8–60.0)	10.0 (5.0–20.0)	
Localized Symptoms				
Erythema	38 (37.6%)	5 (35.7%)	33 (37.9%)	0.562
Swelling	19 (18.8%)	7 (50.0%)	12 (13.8%)	0.004
Skin change	26 (25.7%)	3 (21.4%)	23 (26.4%)	0.489
Warmth	24 (23.8%)	2 (14.3%)	22 (25.3%)	0.300
Mass	48 (47.5%)	7 (50.0%)	41 (47.1%)	0.534
Pain/discomfort	48 (47.5%)	6 (42.9%)	42 (48.3%)	0.466
Number of Localized Symptoms				
(mean ± SD)	2.0 ± 0.8	2.1 ± 0.9	2.0 ± 0.8	0.515
(median, IQR)	2.0 (1.0–3.0)	2.0 (1.8–3.0)	2.0 (1.0–3.0)	
Systemic Symptoms				
Yes	7 (6.9%)	1 (7.1%)	6 (6.9%)	0.660
No	94 (93.1%)	13 (92.9%)	81 (93.1%)	
Previous Mastitis History				
Yes	34 (33.7%)	1 (7.1%)	33 (37.9%)	0.019
No	67 (66.3%)	13 (92.9%)	54 (62.1%)	
Personal History of Cancer				
Yes	1 (1.0%)	0 (0.0%)	1 (1.1%)	0.861
No	100 (99.0%)	14 (100.0%)	86 (98.9%)	
Family History of Breast Cancer				
Yes	3 (3.0%)	0(0.0%)	3 (3.4%)	0.636
No	98 (97.0%)	14 (100.0%)	84 (96.6%)	
Underlying Diseases				
DM/metabolic syndrome	18 (17.8%)	2 (14.3%)	16 (18.4%)	0.646
Immunocompromised	4 (4.0%)	0 (0.0%)	4 (4.6%)	
No	79 (78.2%)	12 (85.7%)	67 (77.0%)	
Mental disorder				
Yes	8 (7.9%)	1 (7.1%)	7 (8.0%)	0.694
No	93 (92.1%)	13 (92.9%)	80 (92.0%)	
Drinking				
Yes	10 (13.2%)	0 (0.0%)	10 (16.1%)	0.113
No	66 (86.6%)	14 (100.0%)	52 (83.9%)	
Smoking				
Yes	4 (5.3%)	0(0.0%)	4 (6.5%)	0.435
No	72 (94.7%)	14 (100.0%)	58 (93.5%)	

IBC = inflammatory breast cancer, SD = standard deviation, IQR = interquartile range, BMI = body mass index, and DM = diabetes mellitus.

**Table 2:** Ultrasound findings of the study participants (*n* = 101).

Variables	Total	IBC	Mastitis	*p*-Value
Bilaterality				
Right	50 (49.5%)	8 (57.1%)	42 (48.3%)	0.780
Left	50 (49.5%)	6 (42.9%)	44 (50.6%)	
Both	1 (1.0%)	0 (0.0%)	1 (1.1%)	
Lesion Type				
Mass	31 (30.7%)	3 (21.4%)	28 (32.2%)	0.319
Non-mass	70 (69.3%)	11 (78.6%)	59 (67.8%)	
Lesion Location				
Central	41 (41.8%)	8 (61.5%)	33 (38.8%)	0.107
Peripheral	57 (58.2%)	5 (38.5%)	52 (61.2%)	
Multiplicity				
Single	75 (74.3%)	11 (78.6%)	64 (73.6%)	0.489
Multiple	26 (25.7%)	3 (21.4%)	23 (26.4%)	
Echogenicity				
Hypoechoic	74 (74.0%)	10 (71.4%)	64 (73.6%)	0.521
Isoechoic	26 (26.0%)	4 (28.6%)	22 (25.3%)	
Hyperechoic	1 (1.0%)	0 (0.0%)	1 (1.1%)	
Echo pattern				
Homogeneous	38 (37.6%)	4 (28.6%)	34 (39.1%)	0.330
Heterogeneous	63 (62.4%)	10 (71.4%)	53 (60.9%)	
Size				
<1/2	61 (60.4%)	5 (35.7%)	56 (64.4%)	0.001
≥1/2	36 (35.6%)	6 (42.9%)	30 (34.5%)	
Whole breast	4 (4.0%)	3 (21.4%)	1 (25.0%)	
Internal Change				
Cystic/Necrotic	30 (29.7%)	1 (7.1%)	29 (33.3%)	0.039
No	71 (70.3%)	13 (92.9%)	58 (66.7%)	
Vascularity				
Minimal/Mild	64 (63.4%)	12 (85.7%)	52 (59.8%)	0.150
Moderate/Severe	28 (27.7%)	2 (14.3%)	26 (29.9%)	
No	9 (8.9%)	0 (0.0%)	9 (10.3%)	
Skin Thickening				
Yes	83 (82.2%)	13 (92.9%)	70 (80.5%)	
No	18 (17.8%)	1 (7.1%)	17 (19.5%)	0.237
Parenchymal Edema				
Yes	89 (88.1%)	14 (100.0%)	75 (86.2%)	0.149
No	12 (11.9%)	0 (0.0%)	12 (13.8%)	
Nipple Inversion				
Yes	17 (16.8%)	4 (28.6%)	13 (14.9%)	0.185
No	84 (83.2%)	10 (71.4%)	74 (85.1%)	
Calcifications				
Yes	3 (3.0%)	1 (7.1%)	2 (2.3%)	0.364
No	98 (97.0%)	13 (92.9%)	85 (97.7%)	
Ductal Change				
Yes	33 (32.7%)	5 (35.7%)	28 (32.2%)	0.507
No	68 (67.3%)	9 (64.3%)	59 (67.8%)	
Lymphadenopathy				
Yes	36 (35.6%)	8 (57.1%)	28 (32.2%)	0.136
Equivocal	42 (41.6%)	5 (35.7%)	37 (42.5%)	
No	23 (22.8%)	1 (7.1%)	22 (25.3%)	

IBC = inflammatory breast cancer.

**Table 3:** Histopathologic results of ultrasound-guided biopsy, suggesting mastitis versus IBC (*n* = 86).

Variables	Frequency (%)
Biopsy-proven benign disease suggesting mastitis (*n* = 62)	
Chronic granulomatous inflammation	31 (50.0%)
Chronic granulomatous inflammation and abscess formation	11 (17.7%)
Inflammation and abscess formation	12 (19.4%)
Inflammation	8 (12.9%)
Biopsy-proven malignancy suggesting IBC (*n* = 14)	
Invasive (*n* = 13)	
Invasive ductal carcinoma	11 (78.6%)
Microinvasive ductal carcinoma	1 (7.1%)
Invasive lobular carcinoma	1 (7.1%)
Non-invasive (*n* = 1)	
Ducal carcinoma in situ	1 (7.1%)

IBC = inflammatory breast cancer.

**Table 4:** Logistic regression analysis of factors associated with inflammatory breast cancer.

Variables	Crude OR	95% CI	Adjusted OR	95% CI	*p*-Value
Age					
(mean ± SD)	1.05	1.01–1.09			
≥40 years	10.20	2.61–39.86	2.76	0.25–30.46	0.407
<40 years	ref. = 1		ref. = 1		
BMI					
(mean ± SD)	1.24	1.04–1.49			
Normal weight	ref. = 1				
Overweight + Obesity	999.99	0.01–999.99			
Menopausal Status					
Premenopause	ref. = 1		ref. = 1		
Postmenopause	9.21	2.69–31.61	6.48	0.40–104.99	0.189
Symptom Onset (days ago)					
(mean ± SD)	1.07	1.03–1.11	1.07	1.02–1.14	0.014
Localized Symptoms					
Swelling	6.25	1.86–21.01	15.24	1.68–138.69	0.016
Previous Mastitis History					
Yes	ref. = 1				
No	7.94	0.99–63.54			
Size (US)					
<1/2	ref. = 1		ref. = 1		
≥1/2	2.24	0.63–7.95	4.49	0.52–38.77	0.761
Whole breast	33.60	2.93–385.91	43.09	0.71–999.99	0.129
Internal Change (US)					
Cystic/Necrotic	ref. = 1				
No	6.90	0.81–52.63			
H-W Chisq *			1.48		0.983

* Hosmer–Lemeshow goodness-of-fit test, Chi-square (*p*-value). OR = odds ratio, CI = confidence interval, SD = standard deviation, ref. = reference, BMI = body mass index, and US = ultrasound.

## Data Availability

The datasets generated and/or analyzed during the current study are not publicly available but are available from the corresponding author upon reasonable request.

## Supplementary Materials

The following supporting information can be downloaded at: https://www.mdpi.com/article/10.3390/life15010005/s1. Table S1. Histopathologic results of invasive malignancy.
