# Supplementary material for: Analysis of Clinically Symptomatic Patients to Differentiate Inflammatory Breast Cancer from Mastitis in Asian Women
Source: Life (Basel). 2024 Dec 24;15(1):5. doi: 10.3390/life15010005 (PMC11766986; doi:10.3390/life15010005)
Supplement: Supplementary file 1 [file life-15-00005-s001.zip › life-3319691-supplementary.pdf]

Table S1. Histopathologic results of invasive malignancy (*n* = 13)

Variables	**Frequency (%)**
Invasive tumor grade	
Well differentiated	0(0.0%)
Moderate differentiated	5(38.5%)
Poorly differentiated	8(61.5%)
Estrogen receptor status	
Positive(+)	7(53.8%)
Negative(-)	6(46.2%)
Progesterone receptor status	
Positive(+)	6(46.2%)
Negative(-)	7(53.8%)
HER2 status	
Positive(+)	8(61.5%)
Negative(-)	5(38.5%)
Ki-67 index	
≤20%	5(38.5%)
>20%	8(61.5%)
Tumor subtype	
HR+/HER2-	2(15.4%)
HR+/HER2+	5(38.5%)
HR-/HER2+	3(23.1%)
HR-/HER2-	3(23.1%)

HR=hormone receptor, HER2=epidermal growth factor receptor 2
